# Neurodegenerative Disease-Associated TDP-43 Fragments Are Extracellularly Secreted with CASA Complex Proteins

**DOI:** 10.3390/cells11030516

**Published:** 2022-02-02

**Authors:** Elena Casarotto, Daisy Sproviero, Eleonora Corridori, Maria Cristina Gagliani, Marta Cozzi, Marta Chierichetti, Riccardo Cristofani, Veronica Ferrari, Mariarita Galbiati, Francesco Mina, Margherita Piccolella, Paola Rusmini, Barbara Tedesco, Stella Gagliardi, Katia Cortese, Cristina Cereda, Angelo Poletti, Valeria Crippa

**Affiliations:** 1 Dipartimento di Scienze Farmacologiche e Biomolecolari (DiSFeB), Department of Excellence 2018–2022, Università degli Studi di Milano, Via Balzaretti 9, 20133 Milano (MI), Italy; elena.casarotto@unimi.it (E.C.); eleonora.corridori@unimi.it (E.C.); marta.cozzi@unimi.it (M.C.); marta.chierichetti@unimi.it (M.C.); riccardo.cristofani@unimi.it (R.C.); veronica.ferrari@unimi.it (V.F.); rita.galbiati@unimi.it (M.G.); francesco.mina@unimi.it (F.M.); margherita.piccolella@unimi.it (M.P.); paola.rusmini@unimi.it (P.R.); barbara.tedesco@unimi.it (B.T.); angelo.poletti@unimi.it (A.P.); 2 Genomic and Post-Genomic Center, IRCCS—Mondino Foundation, via Mondino 2, 27100 Pavia (PV), Italy; daisy.sproviero@mondino.it (D.S.); stella.gagliardi@mondino.it (S.G.); cristina.cereda@asst-fbf-sacco.it (C.C.); 3 Cellular Electron Microscopy Laboratory, Department of Experimental Medicine (DIMES), University of Genoa, Via Antonio de Toni 14, 16132 Genova (GE), Italy; gagliani@unige.it (M.C.G.); katia.cortese@unige.it (K.C.); 4 Unit of Medical Genetics and Neurogenetics, Fondazione IRCCS—Istituto Neurologico Carlo Besta, Via Celoria 11, 20133 Milano (MI), Italy

**Keywords:** amyotrophic lateral sclerosis (ALS), frontotemporal lobar degeneration (FTLD), transactive response DNA-binding protein 43 (TDP-43), extracellular vesicles (EVs), chaperone-assisted selective autophagy (CASA), heat shock protein 70 (HSP70), small heat shock protein B8 (HSPB8), Bcl-2 associated athanogene 3 (BAG3)

## Abstract

Extracellular vesicles (EVs) play a central role in neurodegenerative diseases (NDs) since they may either spread the pathology or contribute to the intracellular protein quality control (PQC) system for the cellular clearance of NDs-associated proteins. Here, we investigated the crosstalk between large (LVs) and small (SVs) EVs and PQC in the disposal of TDP-43 and its FTLD and ALS-associated C-terminal fragments (TDP-35 and TDP-25). By taking advantage of neuronal cells (NSC-34 cells), we demonstrated that both EVs types, but particularly LVs, contained TDP-43, TDP-35 and TDP-25. When the PQC system was inhibited, as it occurs in NDs, we found that TDP-35 and TDP-25 secretion via EVs increased. In line with this observation, we specifically detected TDP-35 in EVs derived from plasma of FTLD patients. Moreover, we demonstrated that both neuronal and plasma-derived EVs transported components of the chaperone-assisted selective autophagy (CASA) complex (HSP70, BAG3 and HSPB8). Neuronal EVs also contained the autophagy-related MAP1LC3B-II protein. Notably, we found that, under PQC inhibition, HSPB8, BAG3 and MAP1LC3B-II secretion paralleled that of TDP-43 species. Taken together, our data highlight the role of EVs, particularly of LVs, in the disposal of disease-associated TDP-43 species, and suggest a possible new role for the CASA complex in NDs.

## 1. Introduction

Neurodegenerative diseases (NDs), such as frontotemporal lobar degeneration (FTLD), Alzheimer’s disease (AD), amyotrophic lateral sclerosis (ALS) and Parkinson’s disease (PD), share several pathological hallmarks. Two of the shared mechanisms are: (i) the progressive neurodegeneration arising in specific neurons and spreading to a more extended area, and (ii) the abnormal formation and accumulation of intracellular inclusions of misfolded proteins [1,2,3,4]. These two aspects of NDs are directly related, since misfolded proteins can be transmitted from one cell to another, contributing to the spreading of the disease [5,6,7,8,9]. In fact, affected cells can release misfolded proteins both as free proteins or after their incorporation into lipid bilayer-delimited particles, called extracellular vesicles (EVs); these EVs can be uptaken by other cells transporting their cargo to them [9].

Cells produce and release two main types of EVs: ectosomes or microvesicles (MVs), directly originating from plasma membrane budding, and exosomes arising from the endosomal trafficking system [10,11]. MVs are larger than exosomes, and therefore they are mainly referred to as large vesicles (LVs), whereas the term small vesicles (SVs) usually refers to exosomes. Both types of EVs carry common, but also specific, protein cargoes [12].

The EVs release of misfolded proteins can be viewed as a protective mechanism for the secreting cell, but it may also negatively affect recipient cells. EVs could assist (or be part of) the intracellular protein quality control (PQC) system in proteostasis surveillance [6,13,14]. PQC system consists of chaperone and co-chaperone proteins, together with the ubiquitin proteasome system (UPS) and autophagy. PQC system components, such as the heat shock protein (HSP) HSP70, are typically found in EVs [12]. Moreover, SVs biogenesis shares important mechanisms with autophagy [13,14], and a new secretory autophagy pathway has recently been identified: the microtubule-associated protein 1 light chain 3 (MAP1LC3B, also known as LC3)-dependent EV loading and secretion (LDELS) [15,16,17].

Due to this Janus activity of EVs on the releasing or on the acceptor cells, understanding the crosstalk between EVs and PQC system in misfolded proteins disposal is crucial in NDs.

Transactive response DNA-binding protein 43 (TDP-43) can be released by EVs [18,19,20,21,22,23,24]. TDP-43 is the main component of pathological aggregates found in the affected cells of half of FTLD and of almost all ALS patients [25,26], as well as of other NDs [3,27,28]. TDP-43, encoded by the *TARDBP* gene, acts as a multifunctional RNA binding protein (RBP) mainly localized in the nucleus; here, TDP-43 regulates RNA transcription and splicing [29]. In pathological conditions, TDP-43 undergoes abnormal cytoplasmic localization and aggregation [30,31]. TDP-43 aggregates are composed of the full-length (FL) 43 kDa protein, and of its C-terminal fragments of 35 (TDP-35) and 25 (TDP-25) kDa [29,30,32], which show differential seeding properties and neurotoxicity [32,33]. TDP-43 aggregates can result from *TARDBP* mutations, post-translational modifications or from stressful events, including an impairment of the PQC system [27,34].

The clearance of FL and fragmented TDP-43 is mediated by both UPS and autophagy, assisted by chaperone and co-chaperone proteins [32,35,36,37,38,39]. Chaperones, such as HSP70, its partners the E3-ubiquitin ligases C-terminus HSP70 interacting protein (CHIP) and its co-chaperones Bcl-2 associated athanogene (BAG) proteins (BAG1 and BAG3), are of particular interest in this context. In fact, the HSP70/CHIP/BAG1 complex favored the disposal of TDP-43, TDP-35 and TDP-25-insoluble species via UPS, whereas the HSP70/BAG3/HSPB8 complex, also known as the chaperone-assisted selective autophagy (CASA) complex, promoted their degradation via autophagy in different ALS cell models [36,37]. Interestingly, BAG3 has been found into SVs [40].

Beside intracellular degradation, EVs have a clear role in FL TDP-43 disposal [18,19]. However, most studies focused only on SVs, without considering LVs in TDP-43 trafficking. For this reason, in this study, we investigated TDP-43 secretion both in LVs and SVs, using EVs derived from immortalized neuronal cells and from plasma of FTLD patients and healthy volunteers. We analyzed all TDP-43 species (FL vs. fragments) and their solubility status (soluble vs. insoluble). Moreover, we mimicked the disease-associated PQC system impairment (with PQC system inhibitors) and we investigated its effect on TDP-43 species secretion. We also analyzed the possible role of HSP70 and its partners in this process. We found that both types of EVs transported insoluble forms of TDP-43, its C-terminal fragments and CASA complex components. The blockage of the PQC system increased the release of these proteins in EVs. These data suggest that both LVs and SVs may have a role in TDP-43 species disposal and that the CASA complex is involved in this process.

## 2. Materials and Methods

### 2.1. Chemicals

Z-Leu-Leu-Leu-al (MG132) (#C2211, Sigma-Aldrich, St. Louis, MO, USA) was used at a dose of 10 μM for 16 h; ammonium chloride (NH_4_Cl) (#EMRO895009, Euroclone, Pero, Italy) was used at a dose of 20 mM for 16 h.

### 2.2. Cell Cultures

Neuroblastoma spinal cord NSC-34 cells, kindly provided by Dr. N.L. Cashman (University of British Columbia, Vancouver, BC, Canada), are routinely cultured in our laboratory [36,41,42]. Cells are maintained at 37 °C in 5% CO_2_ in complete medium (DMEM high glucose medium (#ECB7501L, Euroclone) supplemented with 5% fetal bovine serum (FBS) (#F7524, Sigma-Aldrich), 1 mM l-glutamine (#ECB3004D, Euroclone), penicillin (#31749.04, SERVA Electrophoresis GmbH, Heidelberg, Germany) and streptomycin (#S9137-25G, Sigma-Aldrich).

To perform EVs secretion and isolation, cells (100,000 cells/mL) were seeded in 10 Primo**^®^** TC dishes 150 mm (#ET20150, Euroclone) in 15 mL of complete medium. When cells reached almost 70% of confluence, they were washed with 5 mL of 0.22 μm filter-filtered PBS, and the medium was replaced with 15 mL of exo-free culture medium with or without 10 μM MG132, 20 mM NH_4_Cl or both compounds. Exo-free medium was obtained by centrifuging the complete medium supplemented with 20% FBS at 100,000× *g* at 4 °C for 16 h (in the ultracentrifuge LE-70 (Beckman Coulter, Brea, CA, USA) equipped with the 50.2 TI rotor (Beckman Coulter)). The pellet containing EVs was discarded and the remaining medium was diluted to 5% FBS using DMEM high glucose, added with 1 mM l-glutamine and penicillin-streptomycin. After 16 h of incubation in exo-free medium, the culture medium was collected for EVs isolation, while cells were harvested and centrifuged at 500× *g* for 5 min at 4 °C and the pellet was collected for RNA and protein extraction analysis.

### 2.3. Subjects

FTLD patients were recruited at the IRCCS Mondino Foundation, Pavia (Italy). Plasma was isolated from three blood samples of FTLD patients. Patients were screened for mutations (Sure Select QXT Target Enrichment, Agilent Technology, Santa Clara, CA, USA) and only sporadic patients negative to known mutations were chosen. Three age-matched healthy volunteers free from any pharmacological treatment were recruited at the Immunohematological and Transfusional Service IRCCS Foundation “San Matteo”, Pavia (Italy) and used as healthy controls (ctrl). Subjects participating in the study signed, before being enrolled, an informed consent form approved by the Ethical Committee (Protocol n°20180049077). Movement Disorder Society (MDS) clinical diagnostic criteria were used [43].

### 2.4. EVs Isolation from NSC-34 Culture Medium

EVs isolation was performed according to differential ultracentrifugation method previously used [19], with few changes (Supplementary Figure S1). In details, NSC-34 culture medium was centrifuged at 500× *g* for 10 min at 4 °C (using the centrifuge Heraeus megafuge 16R, Thermo Scientific, Waltham, MA, USA) to remove dead cells and cell debris, and then the supernatant containing EVs was collected and spun at 2000× *g* for 10 min at 4 °C in the same centrifuge. The resulting supernatant was next centrifuged at 20,000× *g* at 4 °C for 2 h (using the centrifuge Avanti J-25 (Beckman Coulter) and the rotor JA-20 (Beckman Coulter)) to collect LVs. The obtained LVs pellet was then washed with 3 mL of filtered PBS and centrifuged at 20,000× *g* for 1 h at 4 °C (with the ultracentrifuge Optima TL (Beckman Coulter) equipped with the rotor TLA 100.3 (Beckman Coulter)) twice to eliminate any trace of non-LVs particles. The supernatant, containing SVs was centrifuged at 100,000× *g* for 1 h at 4 °C (using the ultracentrifuge LE-70 (Beckman Coulter) with the rotor 50.2 TI (Beckman Coulter)) to pellet SVs. The SVs pellet, as for the LVs, was resuspended in 3 mL of filtered PBS and was centrifuged at 100,000× *g* for 1 h at 4 °C (with the ultracentrifuge Optima TL and the rotor TLA 100.3) twice, to eliminate any trace of non-SVs particles. LVs and SVs pellets were resuspended in 20 μL of PBS with protease inhibitors cocktail (#P8340, Sigma-Aldrich). 5 μL of both LVs and SVs suspension was used for NTA analysis and the remaining 15 μL was used for Western blot (WB) analysis.

### 2.5. EVs Isolation from Plasma

Venous blood (5 mL) was collected in sodium citrate tubes from all patients and controls and processed as previously described [19]. Briefly, platelet-free plasma was centrifuged at 20,000× *g* for 1 h. The pellet was washed in 0.22 µm filter-filtered 1× PBS (Sigma-Aldrich, Milan, Italy). LVs supernatant was filtered through a 0.22 µm filter and spun in an Optima MAX-TL Ultracentrifuge at 100,000× *g* for 1 h at 4 °C, and SVs pellet was washed with 1 mL of filtered 1X PBS.

### 2.6. Nanoparticle-Tracking Analysis

LVs and SVs samples were diluted with PBS to an optimal concentration (107–109 particles/mL). Concentration and size analyses were carried out on a Nanosight NS300 (Malvern, UK) with a rate of approximately 30 frames/s. Particle movement videos (60 s/video) were recorded three times per test, and size and mean concentration were analyzed by the NTA software (version 2.2, NanoSight). The results of NTA were presented as the mean of the three tests.

### 2.7. Transmission Electron Microscopy Analysis

EV preparations were resuspended in 20 μL PBS (pH 7.4) and fixed by adding an equal volume of 2% paraformaldehyde in 0.1 mol/L phosphate buffer (pH 7.4). EVs were then adsorbed for 20 min to Formvar–carbon-coated copper grids by floating the grids on 5 μL drops on parafilm. Subsequently, grids were rinsed in PBS and negatively stained with 2% uranyl acetate for 5 min at RT [44]. Stained grids were embedded in 2.5% methylcellulose for improved preservation and air dried before examination. Electron micrographs were taken at Hitachi TEM microscope (HT7800 series, Tokyo, Japan) equipped with Megaview G3 digital camera and Radius software (EMSIS, Muenster, Germany).

### 2.8. Western Blot

Both cell and EV pellets were lysed in RIPA buffer (0.15 M NaCl, 0.8% sodium deoxycholate, 100 μM sodium orthovanadate, 50 mM NaF, 5 mM sodium iodoacetate, 0.05 M Tris HCl (pH 7.7), 10 mM EDTA (pH 8), 0.08% SDS and Triton X-100 (#X100, Sigma-Aldrich) supplemented with protease inhibitors (#04693116001, complete tablets, Roche Diagnostics GmbH, Mannheim, Germany) (for cells, 200 μL per dish was used, whereas, for EVs, 15 μL was used) for 20 min on ice and then slightly sonicated. This is considered as the total RIPA protein extract. Total RIPA extracts were then quantified with the bicinchoninic acid assay (#PRTD1,0500, Cyanagen Reagents for Molecular Biology, Bologna, Italy). Fifteen μg of proteins was loaded on 12% SDS-polyacrylamide gel, resolved by electrophoresis and transferred to nitrocellulose membrane (#10600003, Amersham^TM^ Protran^TM^ Premium 0.45 μm NC, Cytiva, Thermo Fisher Scientific, Waltham, MA, USA) with the Trans-Blot**^®^** Turbo™ transfer system (Bio-Rad Laboratories, (Bio-Rad, Hercules, CA, USA).

To separate RIPA-soluble and RIPA-insoluble fractions, 15 µg of total RIPA extract was centrifuged at 16,000× *g* for 20 min at 4 °C. The supernatant (corresponding to the RIPA-soluble fractions) was transferred in a new tube with 4x sample buffer (0.2 M Trizma base (#T1503-1KG, Sigma-Aldrich) 5% glycerol 60%, 4% SDS 20% and 2% beta-Mercaptoethanol) and incubated at 95 °C for 10 min. Pellets (corresponding to RIPA-insoluble fraction) were washed with filtered PBS, centrifuged at 16,000× *g* at 4 °C for 20 min, resuspended in 15 µL of 4× sample buffer and incubated at 95 °C for 10 min. RIPA-soluble and RIPA-insoluble samples were then analyzed by WB as described for total RIPA extracts.

Nitrocellulose membranes were incubated for 1 h in a blocking solution (1× TBST (20 mM Trizma base, 140 mM NaCl (pH 7.6) and 0.01% Tween 20 (#P1379, Sigma-Aldrich)) with 5% nonfat dried milk powder (A0830,0500, PanReac AppliChem ITW Reagents, Darmstadt, Germany), and then incubated overnight with the specific primary antibody (Supplementary Figure S2) diluted in the same blocking solution. After incubation, blots were washed 3 times with 1× TBST and then incubated for 1 h with peroxidase-conjugated secondary antibodies (Supplementary Figure S2). After the incubation with the secondary antibody, membranes were washed again 3 times with 1× TBST and 1 time with distilled water and, then, chemiluminescent signals were detected using the Westar ETA C ULTRA ECL Western blotting substrate (#XLS075,0100, Cyanagen Reagents for Molecular Biology) and the Chemidoc XRS System (#1708265, Bio-Rad Laboratories). Quantification analysis was performed using Image Lab Software, version 6.0.1 (Bio-Rad Laboratories).

The relative abundance of the TDP-43 species in cells, LVs and SVs was calculated as the mean ratio between the optical density of a single TDP-43 species and the optical densities of all the immunoreactive TDP-43 species detected in the cell, LV or SV lanes of each independent biological sample (n = 3).

For the analysis of the modulation of TDP-43 species and PQC system components in the presence of treatments with MG132 and/or NH_4_Cl, the optical density of the target protein was divided by the optical density of the loading control protein (histone H3 for cells and SVs, and integrin β1 for LVs). Results were then expressed as relative optical densities ratio between the normalized optical density of each independent biological sample (n = 3) and the mean normalized optical densities of the corresponding untreated (control) samples.

### 2.9. Statistical Analysis

Data are presented as mean ± standard deviation (SD) of n = 3 biological samples. PRISM (version 5) software (GraphPad Software, LaJolla, CA, USA) was used for statistical analysis. For WB, Student’s *t*-test was used. For NTA, Welch’s *t*-test was performed. *p* value < 0.05 was considered statistically significant.

## 3. Results and Discussions

In the present study, we investigated the role of EVs in the secretion of TDP-43 and evaluated a possible role of the CASA complex in this process. We considered all EVs, both SVs for which TDP-43 secretion has already been described [6] and LVs. Indeed, despite the fact that plasma-derived LVs from ALS patients contain FL and fragmented TDP-43 [19], their involvement in TDP-43 trafficking is still unknown. Our first aim was to identify whether TDP-43 and its disease-associated C-terminal fragments are differently secreted from neuronal cells. Secondly, we aimed to clarify the possible interplay between the PQC system and EVs in TDP-43 extracellular release. In particular, we focused on the possible role of HSP70 co-chaperones, BAG1 and HSPB8-BAG3. Finally, by comparing plasma-derived LVs and SVs obtained from FTLD patients and healthy volunteers, we evaluated if any specific TDP-43 signature could be present.

For these purposes, we used EVs released by an immortalized neuronal cell line (NSC-34 cells) and FTLD and control plasma-derived LVs and SVs. NSC-34 cells are characterized by a marked neuronal phenotype achieved by spontaneous differentiation (Supplementary Figure S3) and we routinely use them as a “*bona fide*” model to evaluate TDP-43 pathology and the activity of the PQC system [36,37,41,45]. NSC-34 cells also served as a reliable model for EVs isolation and analysis [46,47]. Given the large numbers of cells needed for the analysis of subsets of size exclusion-purified EVs, NSC-34 cells were not further differentiated with other specific reagents.

### 3.1. NSC-34 Cells-Secreted LVs and SVs Were Enriched in Insoluble TDP-43 Species That Were Almost Absent in Cells

**Figure 1:**
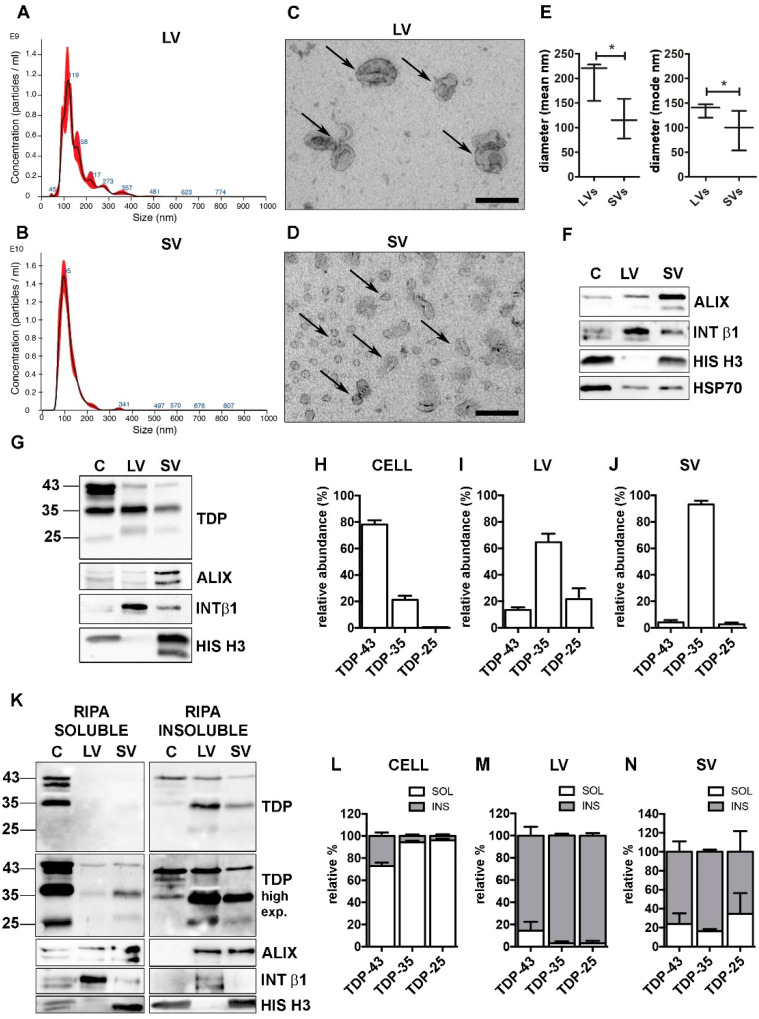
TDP-43 species were differently distributed in cells, LVs and SVs. (**A**,**B**) Representative nanoparticle-tracking analysis (NTA) distribution of LVs (**A**) and SVs (**B**) extracted from NSC-34 cells. (**A**,**B**) *x*-axis = vesicles dimension, expressed in nm; *y*-axis = vesicles concentration, expressed as number of particles/mL. (**C**,**D**) Representative transmission electron microscopy (TEM) of NSC-34 cells-derived LVs (**C**) and SVs (**D**). Scale bar: 200 nm. (**E**) The graph represents the mean (left graph) and the mode (right graph) size ± SD of LVs (200.9 ± 40.7 nm; 136.1 ± 14.1 nm, respectively) and SVs (117.2 ± 40.3 nm; 96.0 ± 40.5 nm, respectively) of n = 3 biological replicates analyzed by NTA (* *p* < 0.05, unpaired one-tailed *t*-test). (**F**) Representative WB analysis of EVs markers in NSC-34-derived LVs and SVs samples. (**G**–**J**) WB analysis of TDP-43, TDP-35 and TDP-25 in total (i.e., soluble + insoluble) RIPA-extracted proteins from NSC-34 cells (C), LVs and SVs released in basal conditions (**G**) and relative quantifications (**H**–**J**). The bar graphs represent the relative abundance of TDP-43, TDP-35 and TDP-25 in cells (**H**), LVs (**I**) and SVs (**J**), expressed as mean [(optical density of a single TDP-43 species/optical densities of all the immunoreactive TDP-43 species) × 100] ± SD of n = 3 biological replicates. (**K**–**N**) WB analyses of RIPA-soluble (left-panels) and RIPA-insoluble (right-panels) fractions of NSC-34 cells, LVs and SVs (**K**) and relative quantifications (**L–N**). The bar graphs represent the relative abundance of each TDP-43 species in the soluble or insoluble fraction of cells (**L**), LVs (**M**) and SVs (**N**), expressed as [(optical density of soluble or insoluble/optical densities of soluble + insoluble TDP) × 100] ± SD of n = 3 biological replicates. (**L**) Cells: TDP-43, 72.8 ± 5.4% soluble; TDP-35, 94.3 ± 2.4% soluble; TDP-25, 96.0 ± 2.7% soluble. (**M**) LVs: TDP-43, 85.6 ± 13.8% insoluble; TDP-35, 96.9 ± 3.0% insoluble; TDP-25, 96.9 ± 3.8% insoluble. (**N**) SVs: TDP-43, 76.0 ± 19.1% insoluble; TDP-35, 83.7 ± 3.8% insoluble; TDP-25, 65.4 ± 7.8% insoluble.

First, we isolated LVs and SVs (Supplementary Figure S1) from the media of NSC-34 cells grown in physiological conditions, and characterized them according to minimal information for studies of extracellular vesicles 2018 (MISEV-2018) guidelines [12]. Nanoparticle tracking analysis (NTA) was used to identify the EVs dimension and concentration. As shown in Figure 1A (LVs), Figure 1B (SVs) and Figure 1E (LVs and SVs relative quantifications), the size of LVs and SVs was statistically different, and in line with that found in transmission electron microscopy (TEM) analysis (LVs, Figure 1C; SVs, Figure 1D).

LVs and SVs were also characterized by different markers detected by WB analysis (Figure 1F). LVs were enriched in the transmembrane plasma membrane integrin β1 (INT β1), whereas SVs were enriched in the accessory regulatory protein of the endolysosomal system ALG-2-interacting protein X (ALIX) [12]. Moreover, LVs were negative, whereas SVs were positive for the nuclear histone H3 protein [48]. Both types of EVs contained the cytosolic marker HSP70. Despite these substantial differences, the two populations partially overlapped and we could not completely exclude that intermediate-sized vesicles were present in both preparations, since a faint immunoreactivity for ALIX and INT β1 was also detected in LVs and SVs, respectively (Figure 1F).

Purified LVs and SVs were then analyzed for their specific TDP-43 content in relation to the levels of TDP-43 species present in the original cell extract. To identify the neurotoxic C-terminal TDP-43 fragments, we selected a specific antibody against the unstructured TDP-43 domain located in the C-terminus (see Supplementary Figure S2 for details). The results are shown in Figure 1G, and relative quantifications in Figure 1H–J.

We analyzed the relative abundance of each TDP-43 species in each type of sample (i.e., cells, LV or SV; see Section 2 for details). Cell lysate used in the WB analysis corresponded to approximately 0.1% of the whole lysate, whereas LV and SV lysates represented 40% and 50%, respectively, of their total extracted proteins.

Figure 1G showed that the TDP-43 immunoreactivity was mainly associated to the FL protein of ~43 kDa (TDP-43), whereas the TDP-35 fragment was poorly represented, and almost undetectable levels were found associated to the TDP-25 fragment, in cells. On the contrary, the immunoreactivity of the TDP-35 fragment was particularly elevated in both LVs and SVs, which were almost lacking the FL TDP-43 and TDP-25 fragment. In line with this observation, we found that, in cells, FL TDP-43 accounted for approximately 80% of the total TDP-43 detected species, followed by TDP-35 (~20%), whereas TDP-25 made up less than 1% of the total TDP-43 species (Figure 1H). A completely different pattern of TDP-43 species distribution was observed in both LVs and SVs; in fact, TDP-35 was the most abundant species in LVs, representing 65% of the total TDP-43 species. The TDP-25 fragment accounted for ~20%, and FL TDP-43 only for ~15% of the total TDP-43 species in LVs. SVs contained a very large amount of TDP-35 (~93%) and only a very small percentage of both TDP-43 (~5%) and TDP-25 (~2%) (Figure 1I,J).

We further investigated RIPA-soluble and -insoluble fractions in our protein samples. The results are reported in Figure 1K (WB of RIPA-soluble and RIPA-insoluble fraction), 1L (WB relative quantification of cells), 1M (WB relative quantification of LVs) and 1N (WB relative quantification of SVs). WB analyses showed that intracellular TDP-43-positive species were largely detected in the RIPA-soluble fraction, whereas TDP-43-secreted forms were almost completely insoluble. These data are in line with a previous report [18] demonstrating that TDP-43 is released in its insoluble form in EVs. In addition, we proved that the insoluble FL TDP-43 protein level was extremely low in LVs and SVs compared to the TDP-35 insoluble protein level. It must be noted that EVs released in a basal condition were particularly enriched in TDP-43 insoluble species, which were almost absent in the secreting cells. Therefore, the secretory pathway plays a relevant role in the disposal of insoluble TDP-43 species normally formed in cells in basal conditions.

### 3.2. EVs Contained PQC System Components Involved in the Intracellular Clearance of Insoluble TDP-43 Species

FL TDP-43 and its C-terminal TDP-43 fragments are cleared mainly via UPS and partly via autophagy, supported by the activity of HSP70 and its co-chaperones, BAG1 and BAG3 [35,36,37,49]. EVs transported HSP70 (Figure 1F) and all TDP-43 species (Figure 1G,K); hence, we wondered whether HSP70 co-chaperones were involved in the EVs secretory pathway.

**Figure 2:**
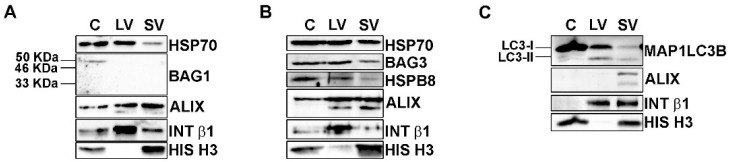
EVs contained HSP70-associated PQC system components. (**A**–**C**) Representative WB analysis of cellular, LVs and SVs samples derived from NSC-34 cells grown in basal conditions showed the presence of HSP70, BAG3, HSPB8 and MAP1LC3B proteins. (**A**) WB showed HSP70 in all samples, whereas its co-chaperone BAG1 was not detected in EVs. (**B**) HSP70, BAG3 and its partner HSPB8 were present in all samples. (**C**) WB analysis showed that cells are enriched in the MAP1LC3B-I form, whereas EVs contained both the MAP1LC3B-I and its lipidated form, MAP1LC3B-II.

The total protein extracts of NSC-34 cells and of their released LVs and SVs were then analyzed by WB (Figure 2).

BAG1-immunoreactivity (Figure 2A) was present with a band of 50 kDa, probably representing the nuclear BAG1 isoform (BAG-1L) [50], only in the whole cell lysate but not in EVs. On the contrary, all samples were immunoreactive for BAG3 and its partner HSPB8 (Figure 2B). Therefore, BAG1 is not secreted, whereas BAG3 and HSPB8 are released in both types of EVs, suggesting that the HSP70 status is preferentially associated with its autophagic route. In fact, HSPB8 and BAG3, together with HSP70, are members of the CASA complex, which is responsible for misfolded proteins recognition and transport to autophagosomes for their disposal via autophagy [51,52,53]. In this context, the loading of the CASA complex-bound substrate into autophagosome is mediated by the SQSTM1/p62 autophagy receptor, but it requires the formation of a lipidated active form of MAP1LC3B (MAP1LC3B-II) that is anchored to the autophagosome membrane. As stated above, MAP1LC3B-II has been proven to be a crucial component of the newly discovered LDELS [15,16,17], where, together with other proteins, it seems to specifically recognize and incorporate cargoes, particularly RBPs, into EVs [17]. We thus analyzed whether our vesicles contain MAP1LC3B-II. We found high levels of the precursor MAP1LC3B-I protein and very low levels of the lipidated MAP1LC3B-II protein in cells (Figure 2C). Interestingly, all EVs were positive for the MAP1LC3B-I form and also enriched in the MAP1LC3B-II form when compared to the whole cell lysates. This result indicates that LDELS secretion is active and could physiologically release EVs of different sizes enriched in FL TDP-43 and its C-terminal fragments. In this context, since EVs also contained HSP70, BAG3 and its partner HSPB8, we hypothesized that the CASA complex could take part in LDELS secretion, targeting substrates of MAP1LC3B-II for EVs secretion. Interestingly, we found HSP70, BAG3 and HSPB8 not only in SVs but also in LVs, thus suggesting that the cargoes and the CASA complex could also be secreted directly from the plasma membrane. In this case, HSPB8 may have a role in EVs secretion, since it directly interacts with the inner leaflet of the plasma membrane [54].

### 3.3. UPS Inhibition Increased The LVs Secretion of TDP-43 Species

**Figure 3:**
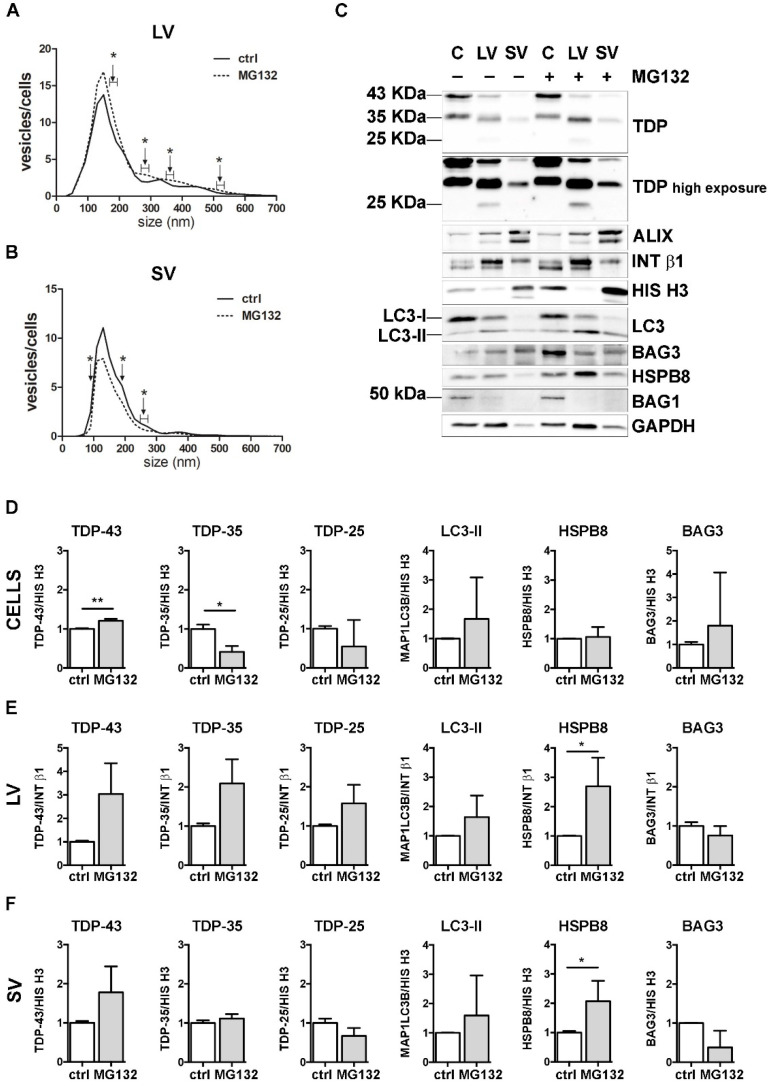
The effect of UPS inhibition on the EVs number and protein content. (**A**,**B**) NTA distribution of LVs (**A**) and SVs (**B**) isolated from NSC-34 cells treated overnight (o/n, 16 h) with MG132 (10 μM). The graphs represent the mean of n = 3 biological replicates; *x*-axis = vesicles dimension, expressed in nm; *y*-axis = vesicles concentration, expressed as ratio between the number of vesicles and the number of secreting cells. The data show that MG132 treatment statistically increased the secretion of 170–190, 270–290, 350–370, 510–530 and 710–730 nm large vesicles (**A**), whereas it statistically reduced the secretion of 90, 190 and 250–270 nm small vesicles (**B**) (* *p* < 0.05, Welch’s *t*-test). (**C**–**F**) WB analysis (**C**) and relative quantifications (**D**–**F**) of total RIPA-extracted proteins from NSC-34 cells, LVs and SVs, untreated or treated o/n with MG132. TDP-43 species, EVs markers and some PQC system members of interest (MAP1LC3B-II, BAG3, HSPB8, BAG1) have been analyzed. Bar graphs represent the mean optical density ± SD of a specific protein normalized on the optical density of the internal housekeeping protein (HIS H3 for cells and SVs; INT β1 for LVs) and reported in comparison to the corresponding untreated sample (ctrl) of three biological replicates (* *p* < 0.05, ** *p* < 0.01, unpaired one-tailed *t*-test).

Then, we investigated the possible crosstalk between the PQC system and EVs in the secretion of FL TDP-43 and its C-terminal fragments. For this purpose, we selectively inhibited UPS, autophagy or both degradative systems in NSC-34 cells and analyzed their EVs release. We first analyzed the effect of the UPS inhibitor MG132 on cells and EVs (Figure 3).

NTA analyses showed that specific subpopulations of LVs were significantly increased after MG132 treatment, compared to control untreated (ctrl) cells (Figure 3A). On the contrary, the number of SVs released by MG132-treated cells was lower than that observed for control cells (Figure 3B).

We next analyzed the effect of UPS blockage on TDP-43 secretion in LVs and SVs released from cells treated or not treated with MG132. Representative WB analyses and TDP-43 species relative quantifications showed that UPS blockage increased the FL TDP-43 form and decreased the TDP-35 fragment, but not the TDP-25 fragment in cells (Figure 3C,D). LVs released upon UPS inhibition were enriched in all TDP-43 species, with a threefold increase in FL TDP-43 and a twofold increase in TDP-35, without reaching statistical significance (Figure 3C,E). SVs did not show any difference in TDP-43 species between the basal condition and after UPS inhibition (Figure 3C,F).

Finally, we analyzed the effect of UPS inhibition on MAP1LC3B, HSPB8 and BAG3. In line with published results [55,56,57], MG132 treatment statistically increased *HspB8* and *Bag3* mRNA and protein levels and led to the conversion of the MAP1LC3B-I protein to MAP1LC3B-II, suggesting that UPS blockage increases autophagy as a compensatory mechanism (Supplementary Figure S4). Regarding EVs, the MAP1LC3B-II signal was slightly raised in all MG132-treated samples compared to control samples (Figure 3C and relative quantifications in Figure 3E,F). Moreover, we found that UPS inhibition statistically increased HSPB8 secretion in both types of EVs, whereas BAG3 secretion was not affected (Figure 3C and relative quantifications in Figure 3E,F). Notably, the HSPB8-BAG3 complex is formed by two HSPB8 molecules *per* BAG3 molecule [58], and thus it is possible that the BAG3-related signal did not increase like that of HSPB8. In addition, it is also possible that HSPB8 acts independently of BAG3. BAG1 levels were not affected by UPS inhibition (Figure 3C).

These data indicate that UPS inhibition slightly affected the global release of EVs, by increasing LVs and reducing SVs. Upon UPS blockage, FL TDP-43 accumulated in cells, but it was also released in EVs. On the contrary, TDP-35 levels decreased in cells, but slightly increased in LVs. TDP-25 species did not change in any conditions. Taken together, these results confirm that FL TDP-43 is mainly degraded by the UPS, but it can also be re-routed to EVs when the system is overwhelmed or blocked [18]. Moreover, the intracellular reduction in TDP-35 and its parallel increase in LVs upon UPS blockage suggest that UPS inhibition might further enhance the basal secretion of TDP-35. Notably, the TDP-25 fragment is not affected by UPS inhibition, possibly because it could also be re-routed to autophagy for degradation, especially in its insoluble form [36,37], and/or constitutively secreted. Finally, the data obtained with MAP1LC3B-II indicate that LDELS secretion increased in response to UPS blockage. The enrichment in HSPB8 and the TDP-43 C-terminal fragments characterizing EVs supports the hypothesis that HSPB8 (together with BAG3 and the CASA complex) plays a central role in the recognition of the cargo to be inserted and transported into EVs.

### 3.4. Autophagy Blockage Did Not Affect Extracellular TDP-43 Species Release

To analyze the effect of autophagy inhibition, we treated cells with NH_4_Cl, which neutralizes lysosomal pH, inhibiting the degradation of autophagosomes into lysosomes. As in the previous set of experiments, both treated cells and their conditioned media were collected for EVs extraction and analyses.

**Figure 4:**
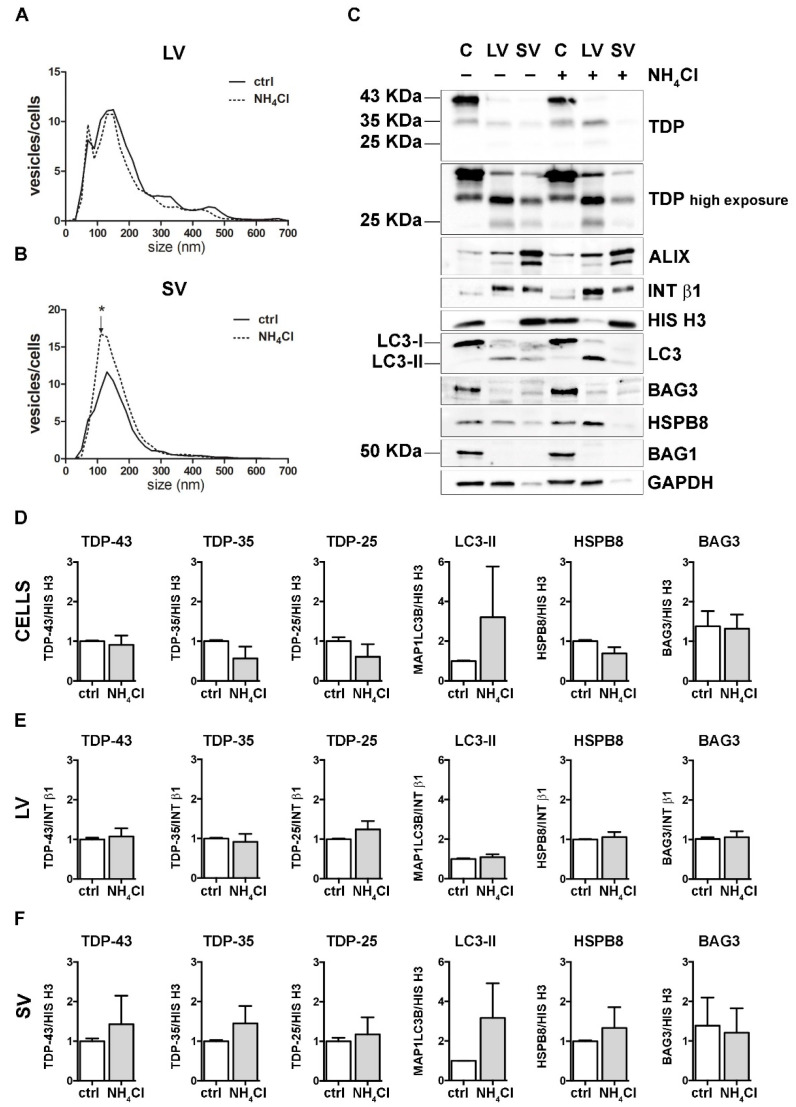
Autophagy inhibition and the modulation of EVs number and protein content. (**A**,**B**) NTA distribution of LVs (**A**) and SVs (**B**) isolated from NSC-34 cells treated o/n with NH_4_Cl (20 mM). The graphs represent the mean of n = 3 biological replicates; *x*-axis = vesicles dimension, expressed in nm; *y*-axis = vesicles concentration, expressed as ratio between the number of vesicles and the number of secreting cells. The data show that NH_4_Cl treatment statistically increased the secretion of 110 nm small vesicles (**B**) (* *p* < 0.05, Welch’s *t*-test). (**C**–**F**) WB analysis (**C**) and relative quantifications (**D**–**F**) of total RIPA-extracted proteins from NSC-34 cells, LVs and SVs, untreated or treated o/n with NH_4_Cl (20 mM). Samples were analyzed for TDP-43 species, MAP1LC3B-II, BAG3, HSPB8, BAG1, ALIX, INT β1 and HIS H3. Bar graphs represent the mean optical density ± SD of a specific protein normalized on the optical density of the internal housekeeping protein (HIS H3 for cells and SVs; Intβ1 for LVs) and reported in comparison to the corresponding untreated sample (ctrl), of three biological replicates.

NH_4_Cl caused a robust accumulation of MAP1LC3B-II in cells, thus confirming the autophagy flux blockage (Figure 4 and Supplementary Figure S4). NTA analyses revealed that autophagy inhibition did not affect LVs release (Figure 4A). Instead, the number of SVs was overall increased upon autophagy blockage, in particular, for SVs of approximately 110 nm in diameter (Figure 4B). No differences were observed between the control and NH_4_Cl-treated samples in the overall protein levels of FL TDP-43, TDP-35 and TDP-25, as well as BAG3, HSPB8 and BAG1, neither in cells, nor in LVs and SVs (Figure 4C and relative quantifications in Figure 4D–F).

These data suggest that autophagy inhibition poorly influenced the overall EVs secretion and did not change the constitutive secretion of different TDP-43 species in NSC-34 cells. Even if our observation diverges from a previous report [18], this could depend on different experimental conditions. Exogenously overexpressed V5-TDP-43 was utilized to investigate the effects of autophagy blockage on TDP-43 release into SVs in the aforementioned work, whereas the endogenous FL TDP-43 and its C-terminal fragments were utilized here to evaluate their release upon different proteotoxic stresses (UPS and autophagy blockage).

### 3.5. The Simultaneous Blockage of UPS and Autophagy Increased the Extracellular LV-Mediated Release of TDP-35, TDP-25 and PQC System Components

**Figure 5:**
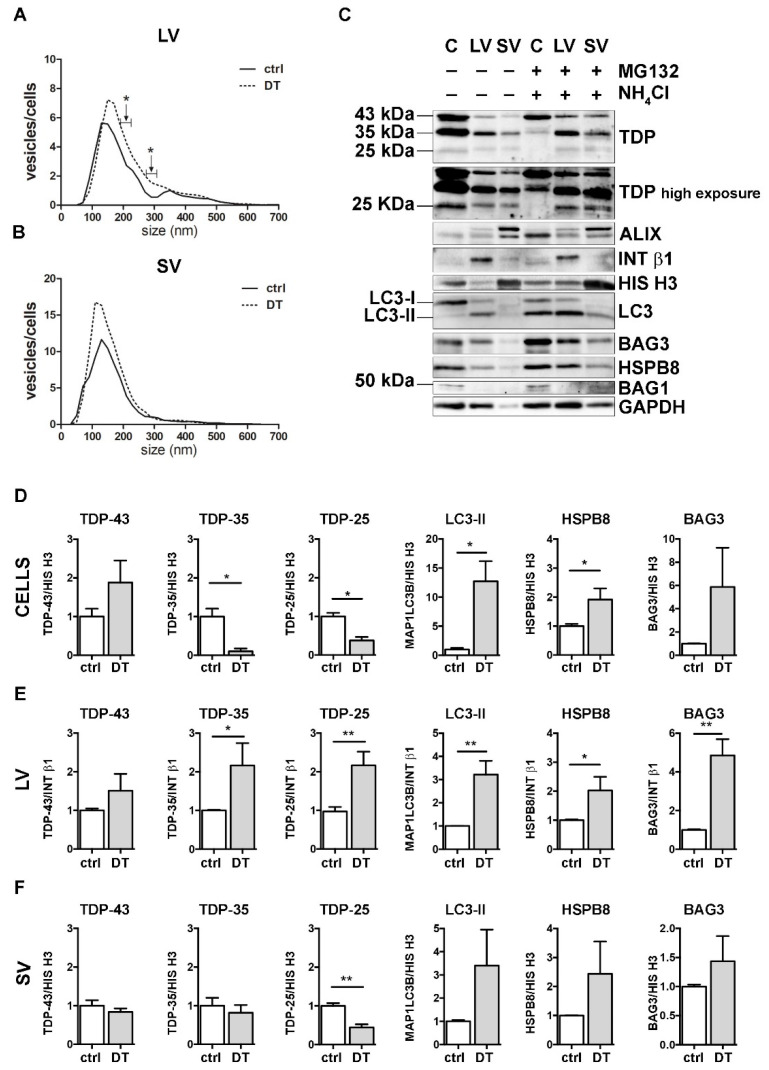
A complete PQC system blockage boosted the extracellular secretion of C-terminal TDP-43 fragments. (**A**,**B**) NTA distribution of LVs (**A**) and SVs (**B**) isolated from NSC-34 cells treated o/n with both MG132 (10 μM) and NH_4_Cl (20 mM) (DT = double-treated samples). Graphs represent the mean of n = 3 biological replicates; *x*-axis = vesicles dimension, expressed in nm; *y*-axis = vesicles concentration, expressed as ratio between the number of vesicles and the number of secreting cells. Data show that PQC blockage statistically increased the secretion of 190–230 and 270–310 nm large vesicles (**A**) (* *p* < 0.05, Welch’s *t*-test). (**C**–**F**) WB analysis (**C**) and relative quantifications (**D**–**F**) of total RIPA-extracted proteins from NSC-34 cells, LVs and SVs, untreated or treated o/n with MG132 (10 μM) and NH_4_Cl (20 mM). Samples were analyzed for TDP-43 species, MAP1LC3B-II, BAG3, HSPB8, BAG1, ALIX, INT β1 and HIS H3. Bar graphs represent the mean optical density ± SD of a specific protein normalized on the optical density of the internal housekeeping protein (HIS H3 for cells and SVs; INT β1 for LVs) and reported in comparison to the corresponding untreated sample (ctrl), for three biological replicates. (* *p* < 0.05, ** *p* < 0.01, unpaired one-tailed *t*-test).

Next, we analyzed the impact of the simultaneous blockage of UPS and autophagy on the EVs release and content. To this purpose, we co-treated cells with MG132 and NH_4_Cl (double-treatment, DT). DT-cells and their released EVs were compared with untreated samples (ctrl) (Figure 5).

DT increased the total number of released LVs/cells, reaching statistical significance between 190–230 nm and between 270–310 nm (Figure 5A). Moreover, treatments resulted in an increase in intracellular FL TDP-43 and in a significant reduction in both TDP-35 and TDP-25 fragments, compared to the ctrl condition (Figure 5C,D). Furthermore, DT-cells showed a robust accumulation of MAP1LC3B-II and an increase in both *HspB8* and *Bag3* mRNA and protein levels (Figure 5C,D, and Supplementary Figure S4). At the same time, the levels of both TDP-35 and TDP-25 almost doubled in DT-LVs (Figure 5C,E). Moreover, DT-LVs showed a threefold increase in MAP1LC3B-II and a significant enhancement of HSPB8 and BAG3 levels when compared to LVs released from untreated cells (Figure 5C,E). SVs released from DT-cells had a lower TDP-25 content and higher MAP1LC3B-II and HSPB8 immunoreactivities compared to those released from control cells (Figure 5C,F).

Taken together, the results showed that the simultaneous blockage of UPS and autophagy enhanced the release of EVs positive to MAP1LC3B-II. This suggests that LDELS secretion could be activated as compensatory mechanism for insoluble species removal. In line with this hypothesis, EVs released when the PQC system was impaired were enriched both in TDP-35 and TDP-25 fragments, whose levels were dramatically reduced in the cell lysates. Notably, the single blockage of UPS or autophagy did not affect the secretion of TDP-25. This supports our previous findings that TDP-25 disposal occurs within the cells either via UPS or autophagy [36,37], and only for a small fraction via EVs. However, when both intracellular degradative systems are hampered, TDP-25 is re-routed to LVs, thus allowing its clearance from cells. Otherwise, TDP-35 clearance appears to be mediated by both UPS and EVs, mainly LVs, and, to a lesser degree, by autophagy. If PQC (in particular UPS) is blocked, TDP-35 secretion is further increased. A specific role in this routing/re-routing of TDP-43 C-terminal fragments into EVs could be exerted by the HSPB8-BAG3 complex, since the expression of the two proteins increased in response to PQC system blockage and both are enriched in EVs released when the PQC system was impaired.

### 3.6. FTLD Patients-Derived EVs Were Enriched in TDP-35 Fragments and Contained CASA Complex Components

**Figure 6:**
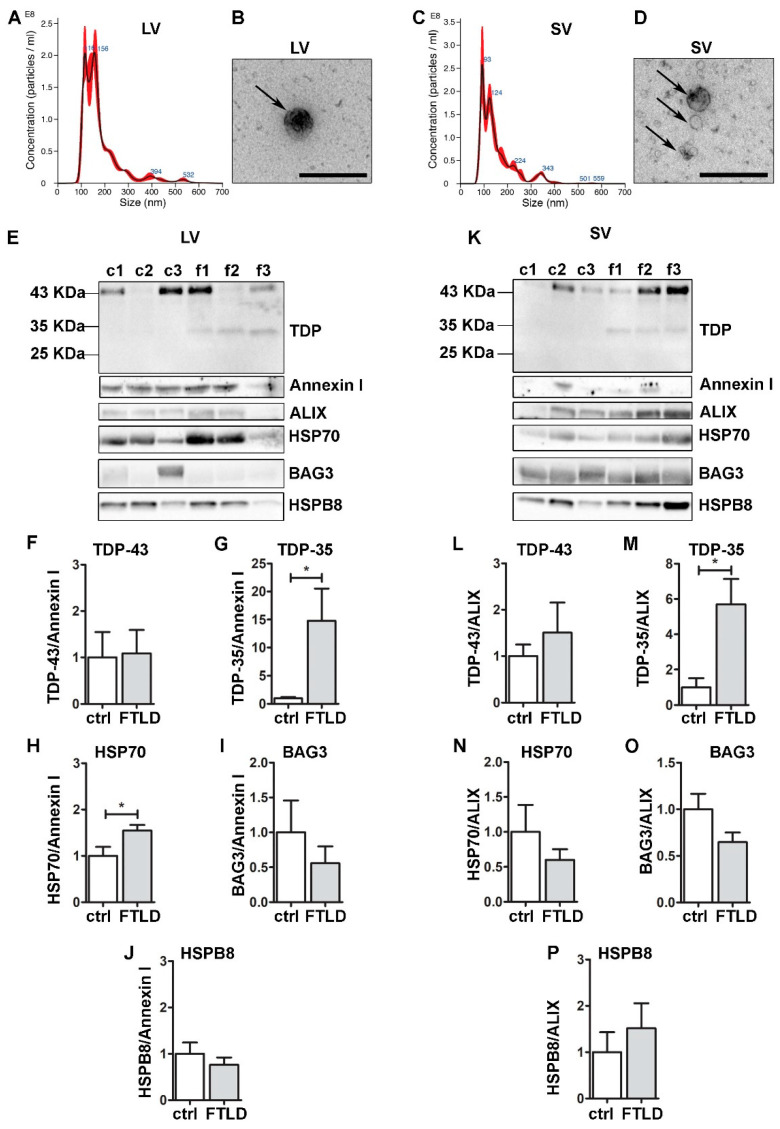
Plasma-derived EVs from FTLD patients are enriched in pathological TDP-35 and contain CASA complex components. Analysis of LVs and SVs from plasma of healthy controls and FTLD patients (n = 3). (**A**,**C**) Representative NTA analysis of LVs (**A**) and SVs (**C**) from plasma of a healthy control. *x*-axis = vesicles dimension, expressed in nm; *y*-axis = vesicles concentration, expressed as number of particles/mL. LV, mean size = 180.2 ± 3.9 nm; SV, mean size = 154.1 ± 1.7 nm. (**B**,**D**) Representative TEM images of LVs (one LV of approximately 200 nm) and SVs (three SVs of approximately 100–150 nm) from plasma of a healthy control (Scale bar: 500 nm). (**E**–**K**) WB and relative quantifications of LVs (**F–J**) and SVs (**L**–**P**) samples from plasma of three healthy controls (c1–c2–c3) and three FTLD (f1–f2–f3) patients. Samples were analyzed for TDP-43 species, Annexin I, ALIX, HSP70, BAG3 and HSPB8. Bar graphs represent the mean optical density ± SD of a specific protein normalized on the optical density of the internal housekeeping protein (annexin I for LVs and ALIX for SVs) and reported as relative to control (ctrl) group, for three biological replicates. (* *p* < 0.05, unpaired one-tailed *t*-test).

To validate the pathophysiological relevance of our observations, we collected and analyzed circulating EVs (both LVs and SVs) obtained from plasma of FTLD patients compared to EVs from healthy donors (ctrl) (Figure 6). LVs and SVs were different in size (Figure 6A–D), as previously demonstrated [19,23]. All EVs were immunoreactive for FL TDP-43, and the CASA complex components HSP70, BAG3 and HSPB8 (Figure 6E–P), reinforcing the hypothesis that the CASA complex has a role in extracellular secretion. No significant difference was observed for BAG3 and HSPB8 between FTLD- and ctrl-derived EVs; instead, the HSP70 signal resulted in being significantly higher in FTLD-derived LVs compared to the ctrl. Data are in accordance with a previous publication, in which HSP70 EVs secretion increased during the course of neurodegeneration in EVs derived from AD and FTLD patients [59].

Interestingly, despite the small number of samples, TDP-35 appeared only in FTLD EVs, both LVs and SVs. This lead result, which will need to be confirmed by analyzing circulating EVs of other FTLD patients, is in line with the observations obtained in NSC-34 cells, where TDP-35 resulted in being the most secreted species and the one whose secretion seems to be boosted when the PQC system is impaired. Given that TDP-43-associated diseases are often related to mutations of PQC system components, as well as PQC system dysfunction, TDP-35 might represent a good biomarker for FTLD.

## 4. Conclusions

In this work, we highlighted the role of EVs, especially of LVs, in the disposal of TDP-43 and its disease-associated C-terminal fragments. In particular, EVs may be used as biomarkers since they transport the TDP-35 fragment, whose secretion specifically increases in pathological situations (i.e., PQC system impairment and FTLD). In this context, EVs seem to work in a cooperative/compensatory manner with the intracellular PQC system. HSPB8-BAG3 secretion appears to increase when the PQC system is impaired and parallels the dynamics of TDP-43 C-terminal fragments. These data suggest that the CASA complex takes part in the recognition of cargoes to be secreted. This process might be associated with MAP1LC3B-II anchored to the multivesicular bodies (MVBs) membrane, which specifically recognizes cargoes to be inserted into MVBs for LDELS secretion. However, a direct budding through the plasma membrane cannot be excluded. In this case, the secretion might be driven by HSPB8, which is capable of binding to the inner leaflet of the plasma membrane.

**Figure 7:**
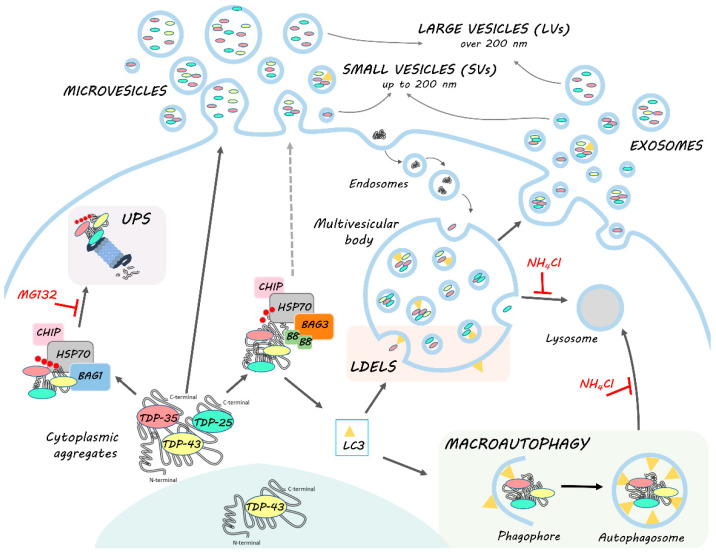
HSPB8-BAG3 involvement in TDP-43 proteostasis and secretion. TDP-43 is mainly localized in the nucleus. In pathological conditions, TDP-43 mislocalizes in the cytoplasm, where it accumulates together with its C-terminal fragments (TDP-35 and TDP-25) into aggregates. The clearance of TDP-43 and its C-terminal fragments occurs primarily via the UPS and autophagy, in cooperation with the PQC system. Among them, HSP70, together with CHIP, recognizes misfolded substrates (such as TDP-43 and its C-terminal fragments) and, by interacting with its co-chaperones BAG1 and BAG3, determines their disposal via UPS or autophagy, respectively. In this context, BAG3 also binds to HSPB8, forming the CASA complex, which assists in the degradation of aberrantly folded substrates mainly via autophagy. Indeed, CASA complex-bound substrates are recognized by MAP1LC3B-II (LC3) protein, associated to the membrane of the phagophore, and thus inserted into the autophagosome for their subsequent lysosomal degradation. However, MAP1LC3B-II can also be associated to the limiting membrane of multivesicular bodies (MVBs), where it recognizes and drives proteins into exosomes for the LC3-dependent extracellular vesicle loading and secretion (LDELS). TDP-43, TDP-35 and TDP-25 proteins bound to CASA complex can then be alternatively driven to autophagy for intracellular degradation or LDELS for secretion. CASA complex might also directly interact with the plasma membrane and bud into LVs. TDP intracellular degradation and extracellular secretion are in balance with each other; in fact, when UPS and lysosomal degradation are blocked, BAG3 and HSPB8 expression is boosted and their secretion, as well as that of MAP1LC3B-II, TDP-35 and TDP-25, increases.

The mechanism we are proposing (Figure 7) paves the way for the understanding of the complex regulation of the EVs dynamics and their pathological role in ALS/FLTD, and contributes to shedding light on the modulation of their secretion. These aspects are particularly relevant, especially because several pharmacological approaches target HSP70 and its partners as a possible treatment of NDs.

## Data Availability

Data are contained within the article or Supplementary Material.

## Supplementary Materials

The following supporting information can be downloaded at: https://www.mdpi.com/article/10.3390/cells11030516/s1, Figure S1: EVs extraction from NSC-34 cells, Figure S2: List of WB antibodies, Figure S3: NSC-34 cells expressed neuronal markers, Figure S4: PQC blockage in NSC-34 cell.
