# Supplementary material for: Neurodegenerative Disease-Associated TDP-43 Fragments Are Extracellularly Secreted with CASA Complex Proteins
Source: Cells. 2022 Feb 2;11(3):516. doi: 10.3390/cells11030516 (PMC8833957; doi:10.3390/cells11030516)
Supplement: Supplementary file 1 [file cells-11-00516-s001.zip › cells-1557480-supplementary.pdf]

Supplementary figure S1. EVs extraction from NSC-34 cells

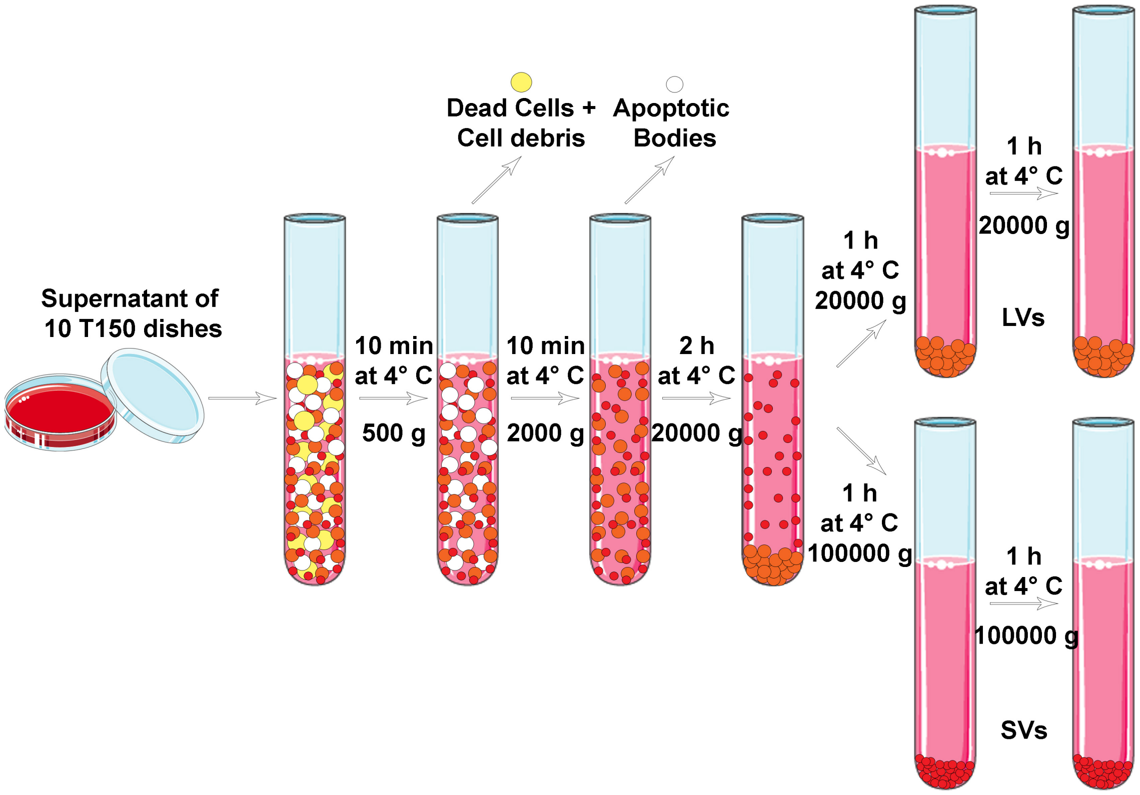

Supplementary figure S2. List of WB antibodies

Antibody	Species	Dilution	Application	Company (Catalog #)
TDP C-Terminal	Rabbit	1:2000	WB	Proteintech (12892-1-AP)
β1-Integrin	Mouse	1:1000	WB	Santa Cruz Biotechnology (sc-374429)
Alix	Rabbit	1:1000	WB	Abcam (ab76608)
GAPDH	Mouse	1:3000	WB	Immunological Science (MAB-10578)
Histone H3	Rabbit	1:10000	WB	Abcam (ab1791)
HSPB8	Rabbit	1:1000	WB	Thermo Fisher (PA5-76780)
BAG3	Rabbit	1:1000	WB	Abcam (ab47124)
BAG1	Rabbit	1:500	WB	Santa Cruz Biotechnology (sc-939)
MAP1LC3B	Rabbit	1:1000	WB	Sigma (L8918)
Anti-Rabbit-HRP conjugated	Goat	1:5000 (TDP C-Term, Alix, HSPB8, BAG3, p62, LC3)<br>1:10000 (Histone H3)	WB	Jackson ImmunoResearch Laboratories (111-035-003)
Anti-Mouse-HRP conjugated	Goat	1:5000 (β1-Integrin)<br>1:10000 (GAPDH)	WB	Jackson ImmunoResearch Laboratories (115-035-003)

Supplementary figure S3.

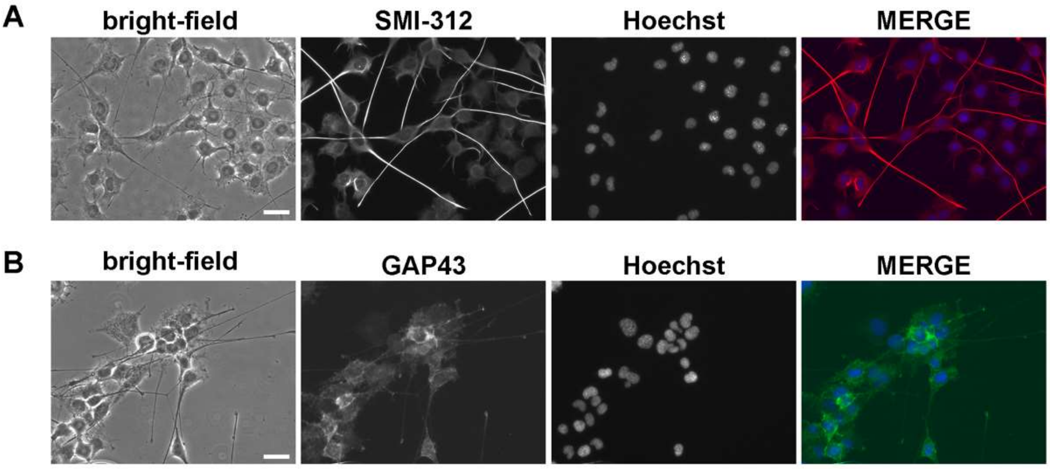

Figure S3. NSC-34 cells expressed neuronal markers. Immunofluorescence analysis of NSC-34 cells. (A) NSC-34 were positive for SMI 312 antibody, which reacts with highly phosphorylated neurofilaments (NF-H and NF-M) and so is a selective marker of mature axons in general. (B) NSC-34 cells expressed the growth associated protein 43 (GAP43), indicator of neurite elongation and synapse formation. Nuclei were stained with Hoechst. Scale bars = 10 mm.

Supplementary figure S4.

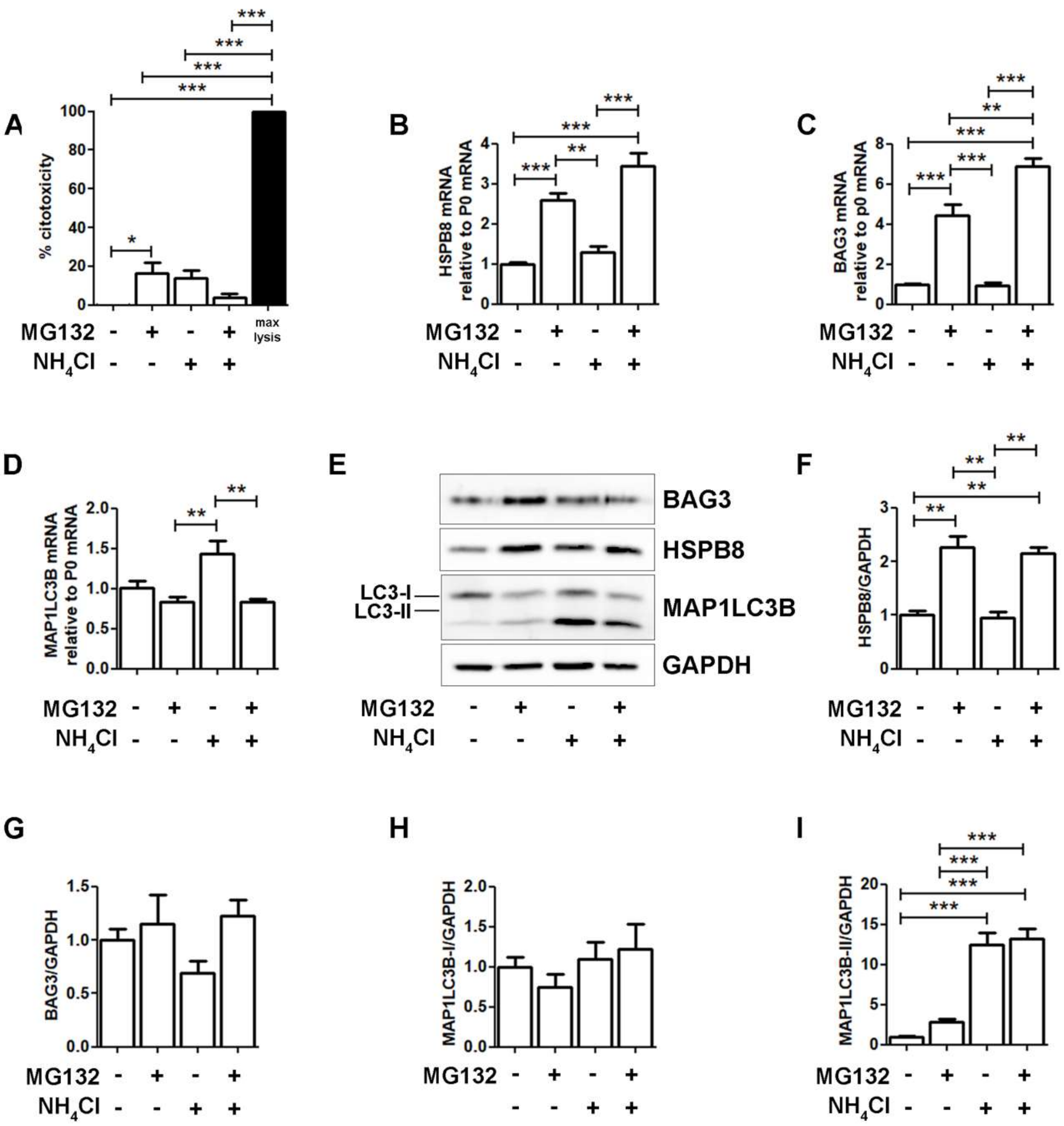

Figure S4. PQC blockage in NSC-34 cells. LDH Assay (A), RT-qPCR (B-D), representative WB analysis (E) and relative quantifications (F-I) of NSC-34 cells untreated or treated o/n with 10 µM of MG132, 20mM NH4CL or both. (A) Bar graph represents the mean percentage of cytotoxicity ± SD of the different treatments in comparison to the control condition of 6 independent biological replicates (*p <0.05, ***p<0.0001, One-way Anova, Bonferroni's Multiple Comparison Test). (B-D) Bar graphs represent the relative fold induction of *HspB8*, *Bag3* and *Map1lc3B* genes normalized with *Rplp0* RNA levels. Data are means ± SD of 4 independent samples (*p < 0.05, ** p < 0.01, One-way Anova, Bonferroni's Multiple Comparison Test). (E) Representative WB analysis of total RIPA-proteins. HSPB8, BAG3 and MAP1LC3B have been analysed. (F-I) Bar graphs represent the mean optical density ± SD of a specific protein normalized on the optical density of GAPDH used as housekeeping protein and reported in comparison to untreated samples. N=3. (*p < 0.05, ** p < 0.01, One-way Anova, Bonferroni's Multiple Comparison Test).

Materials and methods of Supplementary Figures

Immunofluorescence

NSC-34 cells were plated at 35,000 cells/well in 24-well plates containing coverslips, allowed to grow for 48 h and then fixed using a 1:1 solution of 4% paraformaldehyde (PFA) and 4% sucrose in 0.2 N PB (0.06 M KH_2PO_4, 0.31 M Na_2HPO_4; pH 7.4) for 25 min at 37 °C. After blocking with 5% Nonfat dried milk powder and 0.1% Triton X-100, cells were incubated with anti-SMI 312 (dilution 1:200; Sternberger Monoclonals Incorporated) and anti-GAP43 (dilution 1:200; G9264, Sigma-Aldrich) for 1h at room temperature. Cells were washed and incubated for 1h at room temperature with goat anti-mouse Alexa 488 (Life technologies, Thermo Scientific, A-11017; dilution 1:1,000) or goat anti-mouse Alexa 594 (Life technologies, Thermo Scientific, A-11020; dilution 1:1,000) secondary antibodies. After secondary antibody incubation, nuclei were stained with Hoechst (1:2000 in PBS; 33342, Sigma-Aldrich). Images were captured with Axiovert 200 microscope (Zeiss, Oberkochen, Germany) equipped with a photometric CoolSnap CCD camera (Ropper Scientific, Trenton, NJ, USA) using a 20x objective. Images were processed using Metamorph software (Universal Imaging, Downingtown, PA).

LDH Assay

The cytotoxicity of the treatments was determined by measuring the amount of Lactate dehydrogenase (LDH) released in culture supernatant using the CyQUANTUM LDH Cytotoxicity Assay Kit (#C20301, Thermo Fisher Scientific). NSC34 cells were seeded in a 96-well plate at a concentration of 40000 cells/ml. After 24 hours cells were washed with PBS and the medium was changed with Exo-free medium and cells were treated O/N with MG132 (10μM), NH_4Cl (20mM) or both compounds. Lysis buffer was used as positive control. 50 μl of cells supernatant were collected and LDH assays were performed according to the manufacturer's instructions. The absorbance was measured at 490 nm using. N=6. Results were presented as the mean ± SD of 6 independent replicates. Lysis buffer was used as positive control.

RNA extraction and mRNA expression analysis

NSC-34 cells were plated at 150,000 cells/well (4 wells for each condition to be tested; n = 4) in 6-well multiwell plates (#ET3006, Euroclone), allowed to grow for 48 h and then treated with 10 μM MG132 and 20 mM NH_4Cl (together or alone).

24 hours after treatments, the culture medium was collected and discarded, cells were harvested using 1 ml of cold filtered PBS and centrifuged for 5 min at 100 g at 4 °C. The pellet was subsequently resuspended in 300 μl TRI Reagent (#T9424, Sigma-Aldrich) and total RNA isolated according to the manufacturer's instructions. RNA quantification was carried out by absorbance at 260 nm. Total RNA (1 μg) was treated with DNAse I (#AMPD1, Sigma-Aldrich), and reverse transcribed into cDNA using the High-Capacity cDNA Reverse Transcription Kit (#4368813, Thermo Fisher Scientific) according to the manufacturer's protocol. All primers for RT-qPCR, listed below were designed using the Eurofins Genomics software and synthesized by Eurofins Genomics.

Gene	Primer	Sequence (5'->3')
Bag3	forward	ATGGACCTGAGCGATCTCA
	reverse	CACGGGGATGGGGATGTA
HspB8	forward	ATACGTGGAAGTTTCAGGCA
	reverse	TCTCCAAAGGGTGAGTACGG
Map1lc3b	forward	CGTCCTGGACAAGACCAAGT
	reverse	CCATTCACCAGGAGGAAGAA

RplP0	forward	GGTGCCACACTCCATCATCA
	reverse	AGGCCTTGACCTTTTCAGTAAGT

RT-qPCR was performed using the CFX 96 Real Time System (Bio-Rad Laboratories) in 10 µL total volume, using the iTaq SYBR Green Supermix (#1725124, Bio-Rad Laboratories) and 500 nM primers. The PCR cycling conditions used were: 94 °C for 10 minutes, 35 cycles at 94 °C for 15 seconds and 60 °C for 1 minute. At the end of each PCR assay the Melting curve was analysed as a control for specificity. Data were expressed as Ct values and used for the relative quantification of targets using the ΔΔCt calculation. To exclude potential bias because of averaging data were transformed using the equation $2^{-\Delta\Delta Ct}$ to give N-fold changes in gene expression; all statistics were performed with ΔCt values. Each experiment was carried out using 4 independent samples. *Bag3*, *HspB8* and *Map1lc3b* values were then normalized with those of *RplP0*.

Protein expression analysis

NSC34 cells were plated at 90,000 cells/well (3 wells for each condition to be tested; n = 3) in 12-well multiwell plates (#ET3012, Euroclone) and treated as described in the previous paragraph. After the treatments, cells were collected and pelleted as described in Materials and Methods. The pellet was resuspended in 40ul of RIPA buffer containing the Complete protease inhibitor cocktail and processed as described in Materials and Methods. 20 µg of the total RIPA extract of each sample were analysed through WB analysis as in Materials and Methods.
